# Deep learning for hydrocephalus prognosis: Advances, challenges, and future directions: A review

**DOI:** 10.1097/MD.0000000000043082

**Published:** 2025-06-27

**Authors:** Junzhang Huang, Ning Shen, Yuexiang Tan, Yongzhong Tang, Zhendong Ding

**Affiliations:** a Department of General Surgery, Lianjiang Traditional Chinese Medicine Hospital, Lianjiang City, Zhanjiang, Guangdong Province, China; b Department of Anesthesiology, Huadu District People’s Hospital of Guangzhou, Guangzhou, Guangdong Province, China; c The Third School of Clinical Medicine, Southern Medical University, Guangzhou, Guangdong Province, China; d The First School of Clinical Medicine of Guangdong Medical University, Guangzhou, Guangdong Province, China; e SageRAN Technology, Guangzhou, Guangdong Province, China; f Department of Anesthesiology, The Third Xiangya Hospital of Central South University, Changsha, Hunan Province, China; g Postdoctoral Station of Clinical Pharmacology, The Third Xiangya Hospital, Central South University, Changsha, Hunan Province, China.

**Keywords:** deep learning, diagnosis, hydrocephalus, prediction, prognosis

## Abstract

Diagnosis of hydrocephalus involves a careful check of the patient’s history and thorough neurological assessment. The traditional diagnosis has predominantly depended on the professional judgment of physicians based on clinical experience, but with the advancement of precision medicine and individualized treatment, such experience-based methods are no longer sufficient to keep pace with current clinical requirements. To fit this adjustment, the medical community actively devotes itself to data-driven intelligent diagnostic solutions. Building a prognosis prediction model for hydrocephalus has thus become a new focus, among which intelligent prediction systems supported by deep learning offer new technical advantages for clinical diagnosis and treatment decisions. Over the past several years, algorithms of deep learning have demonstrated conspicuous advantages in medical image analysis. Studies revealed that the accuracy rate of the diagnosis of hydrocephalus by magnetic resonance imaging can reach 90% through convolutional neural networks, while their sensitivity and specificity are also better than these of traditional methods. With the extensive use of medical technology in terms of deep learning, its successful use in modeling hydrocephalus prognosis has also drawn extensive attention and recognition from scholars. This review explores the application of deep learning in hydrocephalus diagnosis and prognosis, focusing on image-based, biochemical, and structured data models. Highlighting recent advancements, challenges, and future trajectories, the study emphasizes deep learning’s potential to enhance personalized treatment and improve outcomes.

## 1. Introduction

**Figure 1.**
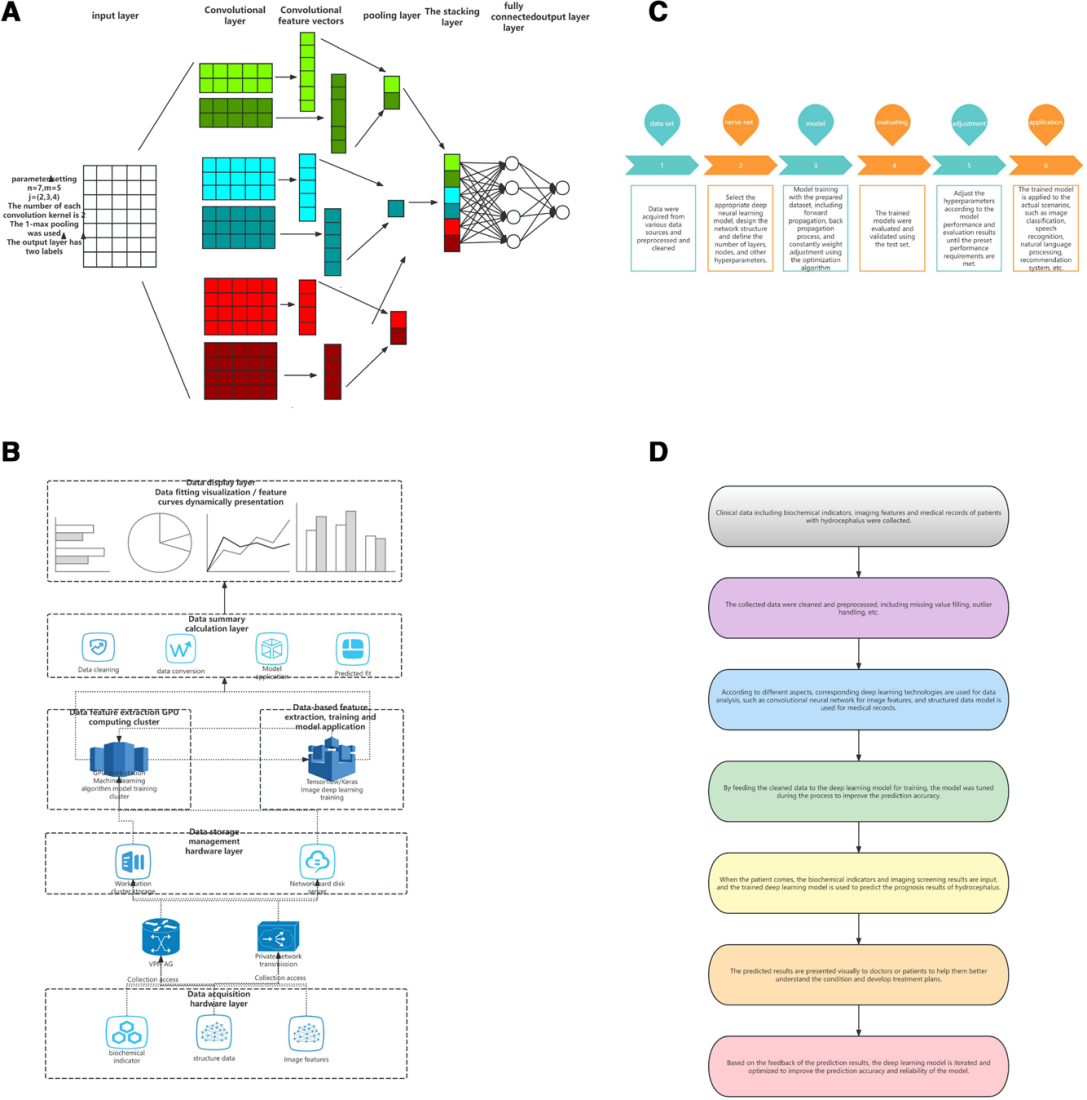
Flow chart of the deep learning model. (A) Internal structural principles of deep learning convolutional neural networks; (B) predictive model of hydrocephalus established based on different features; (C) application of deep learning techniques in clinical research; (D) predictive model processing process of the hydrocephalus data.

Hydrocephalus is a prevalent neurological condition marked by the unusual buildup of cerebrospinal fluid in the brain’s ventricles,^[1]^ causing elevated pressure inside the skull. Without treatment, it can lead to severe outcomes and potentially be fatal.^[2]^ Although radiologists perform excellently in diagnosing hydrocephalus, artificial intelligence, as an initial screening tool, may alleviate their workload and has the potential to match or surpass the diagnostic capabilities of radiologists in the future. Deep learning-driven hydrocephalus prediction models are categorized into 3 distinct types: models that rely on image feature analysis, those predicated on biochemical indicator assessments, and those utilizing structured data analysis. Each category is tailored to harness specific aspects of clinical information, thereby enhancing the predictive accuracy of hydrocephalus outcomes. Our team has carefully analyzed the internal architecture and operational principles of convolutional neural networks in the field of deep learning, utilizing visual aids to enhance understanding, as shown in Fig. 1.

The model based on image features mainly relies on advanced medical imaging technologies such as magnetic resonance imaging and computed tomography to accurately extract and analyze the imaging features of patients with hydrocephalus.^[3]^ Currently, there are studies utilizing automatic segmentation algorithms to automatically measure cerebrospinal fluid and brain volume in children with hydrocephalus.^[4]^ There are also studies indicating that simple linear measurements can serve as an effective alternative to volume analysis for determining ventricular size in patients with idiopathic normal pressure hydrocephalus.^[5]^ In addition, the application of biochemical indicators in the study of hydrocephalus is of great significance for the diagnosis and treatment of the disease. Some literature suggests that biomarkers in cerebrospinal fluid can help improve the management of hydrocephalus in children, providing more accurate diagnosis and treatment efficacy evaluation.^[6]^ The structured data can be used to substantially enhance predictive accuracy in the clinical prediction model. In a research study, a model for the prediction of hydrocephalus was built through the use of structured data, which was able to successfully identify at-risk patients and aid clinicians in early intervention for better prognosis.^[7]^ In spite of the improved diagnosis of hydrocephalus, current methods are not precise enough for individualized treatment. This review aggregates new advances in deep learning approaches in order to meet these limitations.

In summary, deep learning and machine learning techniques provide more objective and accurate tools for the diagnosis and prognosis prediction of hydrocephalus,^[8,9]^ helping clinical doctors make better diagnosis and treatment decisions. Future research should continue to explore the feasibility and effectiveness of these technologies in practical clinical environments.

## 2. The application of deep learning algorithms in the study of hydrocephalus

In recent years, artificial intelligence, more notably, deep learning algorithms, have been extensively used in preventing, controlling, as well as diagnosing infectious diseases like COVID-19.^[10–13]^ In contrast, the use of deep learning algorithms in domestic research involving hydrocephalus has just commenced. Though physicians can visually check the ventricular dilation by undergoing imaging tests in order to make judgments,^[14,15]^ this process might be subject to some limitations. Currently, in contrast with the research advancement in domestic research, the use of deep learning methods in the evaluation of hydrocephalus in foreign countries is thriving. The diagnostic accuracy of hydrocephalus can be enhanced by evaluating the morphology and motion of the endplate.^[8]^

Based on these advances, deep learning algorithms are being used more frequently these days in an attempt to improve the accuracy and efficiency in diagnosing hydrocephalus.^[16]^ With further, it is also possible through the use of deep learning models to accurately differentiate between idiopathic normal pressure hydrocephalus and Alzheimer disease,^[17]^ a matter of vital importance in terms of improving diagnostic accuracy and avoiding misdiagnoses. Yet another related study focuses on the autoencoder model for self-supervised testing time adaptation, performing well in medical image analysis. This technique easily adapts to new data domains by a single test subject involving no source training data, subsequently enhancing the performance of the model across various datasets.^[18]^ These studies demonstrate that with proper dataset augmentation and model design, higher accuracy and robustness in medical image analysis can be achieved by deep learning algorithms.

Within a study, researchers used convolutional neural network training in magnetic resonance images in order to segment ventricles automatically, as well as extract volumetric anatomical information. The results demonstrated that this process was no less specific when identifying cases of hydrocephalus in need of surgery when compared with neuroradiologists, and when externally validated, worked well.^[19]^ Furthermore, the use of deep learning in ventricular segmentation has also been demonstrated as highly efficient and effective. Within a study, researchers designed a systematic automatic ventricular segmentation system. This process not only allows for images of variable scan slice thickness, but also yields highly effective segmentation outcomes in diverse disease conditions.^[20]^ According to current research, deep learning has demonstrated great power in automated hydrocephalus detection, as well as ventricular size measurement, not merely enhancing segmentation precision, efficiency, but also yielding valuable assistance in terms of clinical diagnosis and treatment.

## 3. Establishment of a prognosis prediction model for hydrocephalus

In the design of prognostic models for hydrocephalus, traditional regression analysis is one of the favored methods for researchers in light of its extensive use in medical research and interpretability.^[21–25]^ In the design of clinical predictive models, traditional regression models can well use the data of patients’ risk factors in estimating the likelihood of disease occurrence or future occurrence, thus being extensively used in research in surgery as well as in other medical research. Though traditional regression models are advantaged in terms of interpretability, in some instances, they are susceptible to overfitting, particularly when working with high-dimensional data or small samples. The researchers, therefore, in model development, should exercise special caution in model validation and calibration in ensuring the stability and accuracy of the model across different data sets.

Over the past few years, with the advancement in machine learning technology, random forest, support vector machine, XGBoost, and neural network algorithms are generally used in hydrocephalus prediction and modeling.^[26–29]^ According to studies, research has revealed that, in comparison with traditional algorithms, deep learning algorithms are better in predicting the prognosis of hydrocephalus.^[19,26]^ Deep learning algorithms can deal with high-dimensional and multivariate feature data, while enhancing prediction accuracy, they also involve some degree of “black box” issues and low clinical interpretability. Hence, in studies, several algorithms are usually cross-matched and used in combination with the SHAP value and LIME, for analyzing interpretability in order to facilitate its promotion in clinics.

In artificial intelligence, deep learning has become a potent tool for modeling intricate associations in medical data, specifically for prognosis prediction of hydrocephalus. Convolutional neural networks and recurrent neural networks, as well as their more sophisticated variants such as long short-term memory networks, are increasingly used in interpreting imaging data as well as time-series clinic records, respectively. The models can extract hierarchical feature representations from raw data in a completely automated manner, allowing for more accurate and individualized prognostic estimates. Also, multimodal data consisting of imaging, electronic health records, genetic data, as well as laboratory results, integrated using deep learning frameworks such as multimodal autoencoders, as well as attention-based transformers, promise to enhance model robustness and generalizability. On the ongoing research track, integration of federated learning frameworks with deep learning is also being explored, with objectives spanning from maintaining confidentiality of the patients while propelling large-scale model training from distributed medical data. Deep learning, in all, is driving the limits of hydrocephalus prognosis modeling, promising new avenues of early intervention along with personalized treatment plans.

## 4. Prognostic prediction model of hydrocephalus based on image features

In image-based hydrocephalus prognosis prediction models, deep learning technology excels in performing image segmentation and feature extraction. Image segmentation involves partitioning an image into distinct regions characterized by uniform features. Specifically, in the context of hydrocephalus imaging, this technique is instrumental in demarcating the hydrocephalic regions from the surrounding healthy brain tissue. This differentiation is essential for the model’s construction and the accuracy of its predictions.^[27]^ Convolutional neural networks within the deep learning paradigm are widely recognized for their proficiency in image segmentation tasks.

Feature extraction entails identifying and extracting image characteristics that are indicative of the hydrocephalus condition, providing essential data for diagnostic and prognostic analysis.^[28,29]^ In the context of hydrocephalus imaging, the extracted features may encompass various aspects of the condition, such as the size, morphology, and precise location of the hydrocephalic regions, which are critical for diagnostic and prognostic assessments.^[30,31]^ Deep learning leverages convolutional neural networks and recurrent neural networks as the primary tools for feature extraction from hydrocephalus images. These networks are adept at discerning pertinent features that characterize the condition. Upon completing the processes of image segmentation and feature extraction, the subsequent steps of model training and prediction can be initiated. Deep learning neural network models are capable of learning from these image features to construct a robust prognosis prediction model. Once adequately trained, these models can predict the prognosis and inform the development of tailored treatment plans for new patients presenting with hydrocephalus.

Beyond the techniques previously discussed, the application of deep learning technology extends to the realms of visualization and explainability in the prediction of hydrocephalus prognosis. These aspects are crucial for enhancing the interpretability of the model’s decisions and for fostering greater trust and understanding among medical practitioners.^[32]^ Visualization techniques in deep learning offer a visual representation of the model’s predictions, facilitating a more intuitive comprehension for physicians. Concurrently, explainability technologies demystify the model’s internal workings, promoting greater trust and more effective clinical application. Image-based hydrocephalus prognosis prediction models serve as a decision-support tool for medical professionals,^[33]^ enabling the creation of personalized and precise treatment strategies. This, in turn, can enhance patient outcomes and survival rates by ensuring that treatments are tailored to individual needs. The image-based prediction model for hydrocephalus prognosis has become an important auxiliary decision-making tool for clinical doctors, which helps to develop personalized and accurate treatment plans, thereby improving the treatment effectiveness and survival rate of patients. However, despite the enormous potential of such models in noninvasive and automated analysis, they still have certain limitations in fully revealing the underlying pathophysiological mechanisms of diseases. This also highlights the necessity of introducing other data sources (such as biochemical indicators) to achieve more comprehensive and accurate prognosis assessment.

## 5. A prognosis prediction model of hydrocephalus based on biochemical indicators

Biochemical-based prognosis prediction models focus on the extraction of characteristic features from the biochemical markers of hydrocephalus cases. The extracted features are then matched against the prognosis condition in order to create a prediction model capable of distinguishing the probable outcomes and treatment approaches.^[34]^ Deep learning, in particular, has become enormously popular in recent years, being a preferred solution through its sophisticated abilities in dealing with intricate data patterns.

For example, deep neural networks excel in extracting complex features from biochemical indicator data, a skill set that has made them a highly prized tool in predictive analytics used in prognosis of hydrocephalus. Deep neural networks are defined by their large number of hidden layers able to perform a cascade of complex nonlinear processing of the input data. This depth of architecture allows for better extraction of features, allowing the network to perceive detailed patterns not seen in less deeply structured models. The use of self-guided learning processes allows these networks, in turn, to produce a more detailed and richer set of features, thus greatly improving prognostic prediction model accuracy.

On completion of the feature extraction step, the trained predictive model is ready for deployment in forecasting purposes. For biochemical-based hydrocephalus prognosis models, training, as well as predictive, processes are usually based on machine learning algorithms or statistical methods. Support vector machines, decision trees, and random forests are some of the most frequently used approaches. The importance should be noted here in the selection of proper biochemical indicators in the development of such predictive models.^[35–37]^ Only with the careful selection of biochemical indicators, in combination with proper processing, and analytical methods, accurate prognostic predictions can be achieved. Such a selective, step-by-step process is important in obtaining assured, precise results from the predictive model.

The biochemical hydrocephalus prognosis prediction model provides doctors with a reliable and convenient diagnostic toolkit, improving the accuracy and efficiency of clinical evaluation.^[38]^ By detailed analysis and processing of patient biochemical indicators data, the speed and accuracy of predicting the prognosis of hydrocephalus can be improved. This helps clinical doctors design more personalized treatment plans, thereby providing the best treatment outcomes.

## 6. Prediction model of hydrocephalus prognosis based on structured data

The integration of structured data is particularly crucial in the process of constructing a prognosis prediction model for patients with hydrocephalus using deep learning techniques. Usually, the input data for such models mainly comes from the patient’s basic demographic characteristics (such as age, gender, and medical history), vital signs (such as blood pressure, heart rate, respiratory rate), and routine laboratory test indicators (such as serum protein, white blood cell count, etc).^[39–41]^ In addition, imaging examinations such as magnetic resonance imaging and computed tomography provide important disease characterization information for the model. These data mostly come from clinical examinations, medical records, or monitoring results of medical equipment, which can comprehensively reflect the patient’s health status and disease characteristics, providing important support for subsequent disease prediction and personalized diagnosis and treatment.

The prognosis prediction model for hydrocephalus constructed using structured data has indeed demonstrated certain advantages in model structure design, result interpretation, and validation evaluation. The core purpose of such models is to provide clinical doctors with more accurate, comprehensive, and efficient decision support during the diagnosis and treatment process.^[42–44]^ However, it should be emphasized that there are also many issues worth paying attention to in the practical application of such models.

## 7. Research status and challenges

When developing a hydrocephalus prognosis prediction model using deep learning techniques, we encounter various challenges and constraints. These include the problem of data missingness and quality concerns, the need for careful algorithm selection and optimization, the interpretation of clinical significance, and the critical issues of privacy and security.

Addressing these issues is vital to enhance the predictive capabilities of the model and to ensure that it can provide reliable and clinically relevant insights. Firstly, the integrity and accuracy of data are the foundation of model establishment. In the process of data collection and processing, it is necessary to avoid data loss and input errors as much as possible, ensuring that the data used is true and comprehensive. Secondly, in the actual application process of the model, corresponding strategies should be formulated to reasonably address possible missing data issues.

For example, the prediction threshold of the model can be dynamically adjusted according to the actual situation, or more individual patient characteristics can be introduced to improve the adaptability and prediction performance of the model in complex clinical situations. Finally, patient privacy and data security are equally important aspects that cannot be ignored in the application process of the model. We should strengthen technical protection measures and strictly ensure the security and confidentiality of patient information. This not only respects the rights and interests of patients, but also helps to enhance the trust and acceptance of clinical medical staff and patients towards such predictive models.

The selection and optimization of deep learning algorithms are intricate processes that demand a high level of technical proficiency. It is essential to choose the appropriate deep learning model tailored to specific application contexts and data characteristics. Deep learning models are widely used in the field of engineering,^[45,46]^ but there are still many shortcomings in the medical field, such as the prognosis of hydrocephalus. Accurate predictive capabilities are crucial for enabling medical professionals to intervene with greater precision and timeliness. Furthermore, these capabilities are instrumental in delivering tailored diagnostic and therapeutic strategies to patients, enhancing the personalization and efficacy of healthcare delivery.^[47,48]^ To address this issue, one can employ traditional model optimization methods such as feature selection and feature engineering to refine the model’s performance. Alternatively, leveraging deep learning techniques can further enhance the model’s accuracy by tapping into the power of complex neural networks to learn intricate patterns within the data.

In summary, deep learning technology has demonstrated a new research approach and technical path in the development of prognostic prediction models for hydrocephalus, which is expected to provide strong support for accurate diagnosis and early intervention of hydrocephalus patients. However, the practical application of relevant research still faces many challenges and limitations, and further optimization and validation are needed in terms of model stability, interpretability, and clinical promotion value. In the future, research in this field urgently needs to promote the deep integration of technology and medicine while ensuring scientific rigor, combined with clinical needs, to ensure its effectiveness and safety in real-world scenarios.^[49]^ Table 1 compares and analyzes the application effects of commonly used machine learning algorithms and deep learning models.

**Table 1:** Comparison of machine learning and deep learning algorithm models.

Order number	Model	Field of application	Advantage	Disadvantage
1	Linear regression	Suitable for predicting quantitative variables	Can deal with outliers and noise	Modeling nonlinear relationships was poorly
2	Logistic regression	Applicable to dichotomy problems	Good interpretability and faster computational speed	But for multi-classification problems.
3	Decision tree	Can handle numerical and categorical data	Easy to explain and simple to construct	It is easy to have overfitting and underfitting problems.
4	Random forest	Composed of multiple decision trees	The problem of overfitting and underfitting of decision trees can be solved	Difficulties in dealing with high-dimensional sparse data.
5	Support Vector Machine	Suitable for small-sample, high-dimensional data	Stable performance, can solve the linear, nonlinear problems	Reference tuning is required to achieve the optimal results
6	K-nearest neighbor	Can deal with the nonlinear classification problems	The training process is not required	However, the processing efficiency is low for data with high dimension and large sample size
7	Neural network	Applicable to the nonlinear problems	Can process large-scale data	With a long training time and requires a large amount of computational resources, multiple factors need to be considered in the modeling process
8	Convolutional neural network	Mainly used in image, voice and other fields	Can reduce the number of parameters, while improving the accuracy	The data requirements are relatively high
9	Recurrent neural network	Suitable for serialized data, such as time series, natural language processing, etc	You can retain the historical information	Prone to gradient disappearance or explosion.
10	Long and short-term memory networks	Long and short-term memory networks are mainly used to process the serialized data	The gradient problem in the algorithm can be solved	It is easy to overfit the data and requires appropriate regularization operations to prevent this problem.

## 8. Trend in development

As internet technology advances, the landscape of data sources has become increasingly diverse, encompassing platforms such as social media and data generated by mobile devices. The breadth of these sources introduces new challenges for hydrocephalus prognosis models, as they may be replete with noise and unreliable information. Consequently, in the realm of data processing, there is a growing need for models that exhibit greater intelligence and adaptability. With the progression of deep learning technologies, an increasing number of applications are embracing deep learning models.^[49]^ As computational capabilities continue to enhance, the training and optimization processes for deep learning models will correspondingly become more efficient, allowing for faster and more sophisticated model development.

In certain scenarios, the interpretability of a model outweighs its accuracy. Models that are easily interpretable provide physicians with a clearer understanding of the model’s decision-making processes, which is invaluable for gaining trust and facilitating the integration of AI into clinical practice. As interpretable models evolve, prognosis models will increasingly offer enhanced interpretability. This advancement aids healthcare professionals in gaining a deeper comprehension of the models’ outcomes, thereby refining the precision of medical decision-making. Concurrently, with the advancement of medical technology, personalized medicine is emerging as a pivotal trend in the future of healthcare, promising tailored treatments that are better suited to individual patient needs.^[50–52]^ Personalized medicine will offer patients customized treatment plans that are meticulously crafted to align with their unique individual traits and medical history. This approach ensures that healthcare is not only more targeted but also more effective, as it accounts for the specific genetic, environmental, and lifestyle factors that influence an individual’s health.^[53–57]^

In summary, the further development of prognostic prediction models for hydrocephalus relies on the organic combination of data analysis techniques, algorithm methods, model interpretability, and personalized medical care. Only on this basis can we better adapt to the constantly changing and upgrading needs of modern healthcare, and truly provide more accurate, efficient, and valuable auxiliary support for clinical doctors in the diagnosis and treatment process.

## 9. Limitations and future work

Although deep learning has shown promising application prospects in the diagnosis and prognosis of hydrocephalus, there are still some shortcomings that deserve attention. First of all, the establishment of the model often depends on a large number of high-quality annotation data, but limited by the privacy protection of patients and the low incidence rate of some hydrocephalus subtypes, it is not easy to obtain relevant data. Secondly, differences in patient populations, imaging equipment, or scanning parameters may affect the stability and applicability of the model. Thirdly, the “black box” nature of deep learning models, which lacks clear decision-making basis, also limits their widespread recognition in clinical practice to a certain extent. In addition, most current research still relies mainly on imaging data and lacks the integration of multimodal data (such as genetic information, clinical data, and laboratory indicators), which affects the comprehensiveness of the model and further enhances its clinical value.

Future research can compensate for the shortcomings of current research by developing more stable and reliable models. For example, using transfer learning or data augmentation methods to enable the model to learn effectively even with small amounts of data. In addition, adopting more interpretable model designs can help clinical doctors better understand and apply them.

## Author contributions

**Conceptualization:** Junzhang Huang, Ning Shen, Yuexiang Tan.

**Investigation:** Junzhang Huang, Ning Shen, Yuexiang Tan.

**Supervision:** Yongzhong Tang, Zhendong Ding.

**Validation:** Yongzhong Tang, Zhendong Ding.

**Writing – original draft:** Junzhang Huang, Ning Shen.

**Writing – review & editing:** Yongzhong Tang, Zhendong Ding.
